# VviWRKY24 promotes *β*-damascenone biosynthesis by targeting *VviNCED1* to increase abscisic acid in grape berries

**DOI:** 10.1093/hr/uhaf017

**Published:** 2025-01-15

**Authors:** Yi Wei, Yachen Wang, Xiao Meng, Xuechen Yao, Nongyu Xia, Huimin Zhang, Nan Meng, Changqing Duan, Qiuhong Pan

**Affiliations:** Center for Viticulture and Enology, College of Food Science & Nutritional Engineering, China Agricultural University, Beijing 100083, China; Key Laboratory of Viticulture and Enology, Ministry of Agricultural and Rural Affairs, Beijing 100083, China; Center for Viticulture and Enology, College of Food Science & Nutritional Engineering, China Agricultural University, Beijing 100083, China; Key Laboratory of Viticulture and Enology, Ministry of Agricultural and Rural Affairs, Beijing 100083, China; Center for Viticulture and Enology, College of Food Science & Nutritional Engineering, China Agricultural University, Beijing 100083, China; Key Laboratory of Viticulture and Enology, Ministry of Agricultural and Rural Affairs, Beijing 100083, China; Center for Viticulture and Enology, College of Food Science & Nutritional Engineering, China Agricultural University, Beijing 100083, China; Key Laboratory of Viticulture and Enology, Ministry of Agricultural and Rural Affairs, Beijing 100083, China; Center for Viticulture and Enology, College of Food Science & Nutritional Engineering, China Agricultural University, Beijing 100083, China; Key Laboratory of Viticulture and Enology, Ministry of Agricultural and Rural Affairs, Beijing 100083, China; Center for Viticulture and Enology, College of Food Science & Nutritional Engineering, China Agricultural University, Beijing 100083, China; Key Laboratory of Viticulture and Enology, Ministry of Agricultural and Rural Affairs, Beijing 100083, China; Key Laboratory of Brewing Molecular Engineering of China Light Industry, Beijing Technology and Business University, Beijing 100048, China; Center for Viticulture and Enology, College of Food Science & Nutritional Engineering, China Agricultural University, Beijing 100083, China; Key Laboratory of Viticulture and Enology, Ministry of Agricultural and Rural Affairs, Beijing 100083, China; Center for Viticulture and Enology, College of Food Science & Nutritional Engineering, China Agricultural University, Beijing 100083, China; Key Laboratory of Viticulture and Enology, Ministry of Agricultural and Rural Affairs, Beijing 100083, China

## Abstract

Norisoprenoids, which are produced by the cleavage of various carotenoids, are a class of volatile aroma compounds that widely distributed in plants. In wine, they represent a significant source of floral and fruity aromas. *β*-Damascenone is the most abundant and important norisoprenoid constituent in grape berries (*Vitis vinifera* L.) and wines. However, the regulatory mechanism of *β*-damascenone biosynthesis remains poorly understood. The present study has identified a WRKY transcription factor, VviWRKY24, as a key regulator of *β*-damascenone accumulation in grape berries. The results of overexpression and gene silencing assays in grape leaves, berries, and calli demonstrated that VviWRKY24 altered the flow of norisoprenoid metabolism and influenced the composition ratio of norisoprenoids, particularly enhancing the levels of *β*-damascenone. The results of the RNA-seq, yeast one-hybrid, electrophoretic mobility shift, and dual-luciferase assays provided confirmation that VviWRKY24 promoted abscisic acid (ABA) biosynthesis by directly upregulating the expression of *VviNCED1*. The increase in ABA content resulted in further induction of the expression of *carotenoid cleavage dioxygenase 4B* (*VviCCD4b*) on *β*-damascenone metabolic pathway. These findings elucidate the upstream regulation of ABA and the promotion of ABA on the accumulation of *β*-damascenone in grapes. This study contributes to a novel understanding of the regulatory mechanisms of *β*-damascenone biosynthesis and provides a strategy for improving the aroma quality of grapes and wine.

## Introduction

Wine is the oldest known alcoholic beverage and remains a popular beverage around the world. The aroma of wine serves as an important indicator of its quality. To date, over 800 volatile compounds have been identified in wine [1]. Among them, norisoprenoids represent a class of aroma compounds derived from grape berries (*Vitis vinifera* L.) with a pleasant floral and fruity odor. They are of great consequence with respect to the expression of wine flavor and typicality. The olfactory contributions of different norisoprenoid components are distinct. In grapes and wines of *V. vinifera* species, *β*-damascenone is typically the most abundant of these components [2, 3].

As a branch of isoprenoid metabolic pathway, norisoprenoid biosynthesis commences with the cleavage of carotenoids within the plastid. Under the catalysis of enzymes such as phytoene synthase (PSY), geranylgeranyl diphosphate (GGPP) from 2-methyl-D-erythritol-4-phosphate (MEP) pathway is sequentially converted into phytoene, *ζ*-carotene, lycopene, *β*-carotene, *β*-cryptoxanthin, antheraxanthin, violaxanthin and neoxanthin. These carotenoids can undergo further cleavage by carotenoid cleavage dioxygenase (CCD) to generate norisoprenoids [4]. Both phytoene and *ζ*-carotene can serve as the precursors to geranylacetone, while lycopene can undergo degradation to form 6-methyl-5-hepton-2-one (MHO). *β*-ionone and *β*-cyclocitral are derived from *β*-carotene through the breaking of double bond at different position. *β*-Damascenone, along with other C_13_-norisoprenoids such as trimethyl-dihydronaphthalene (TDN), are considered to be produced by violaxanthin and neoxanthin through a series of enzymatic and non-enzymatic reactions under acidic condition [5, 6].

Violaxanthin and neoxanthin also serve as the precursors to abscisic acid (ABA). They undergo transformation into 9′-*cis*-violaxanthin and 9′-*cis*-neoxanthin, and are further cleaved by 9-*cis*-epoxycarotenoid dioxygenase (NCED), which is the key enzyme in ABA biosynthesis [7]. ABA plays a role in plant developmental processes [8] and stress tolerance [9], including the ability to withstand drought or cold stress. In the case of grapes, ABA plays a pivotal role in the development and maturation of berries [10]. Three NCEDs have been identified in grapes: VviNCED1, VviNCED2, and VviNCED6 [4]. In the majority of grape varieties, the expression of *VviNCED1* is linked to the onset of grape ripening, with a corresponding pattern of ABA accumulation [11].

ABA not only shares the same substrate with *β*-damascenone biosynthesis, but also acts as a hormone to affect the accumulation of norisoprenoids, especially *β*-damascenone, as previously reported in our studies. In two consecutive years of repeated experiments, exogenous ABA treatment prior to véraison was observed to increase the content of free norisoprenoids in grape berries [12]. Another study demonstrated that spraying 1-naphthaleneacetic acid (NAA) at pre-véraison elevated the endogenous ABA levels at véraison, which proved advantageous for the accumulation of *β*-damascenone and TDN in grape berries. Subsequent experiments have corroborated the finding that ABA treatment can enhance the accumulation of *β*-damascenone and TDN in detached grape berries [13]. Furthermore, our findings revealed that the expressions of multiple genes involved in norisoprenoid biosynthesis, including *VviPSY1*, *VviCCD4b*, and *VviNCED1*, were enhanced by ABA [13–15]. Nevertheless, the precise interrelationship among the genes, norisoprenoids and ABA remains to be elucidated.

The transcriptional regulatory mechanism underlying the production of norisoprenoids remains incompletely understood. Several transcription factors (TFs) have been identified as positive or negative regulators of *β*-ionone in *Osmanthus fragrans* [16–20]. To date, only three TFs associated with norisoprenoid synthesis have been identified in fruits. SlMYB75 has been found to enhance the production of non-specific volatile aromas, including norisoprenoids in tomato fruits (*Solanum lycopersicum*). VviWRKY70 and VviMADS4 have been demonstrated to negatively regulate the norisoprenoid accumulation in grape berries [21, 22]. However, none of the aforementioned reports addressed the impact on *β*-damascenone synthesis.

Given that ABA shares the same carotenoid metabolic pathway with *β*-damascenone and exerts an effect on norisoprenoid accumulation as a hormone, we are attempting to identify TFs that may be involved in these processes. In plants, the WRKY TF superfamily represents the second largest family of regulatory proteins involved in stress and development [23]. The DNA-binding domain of WRKY TF is approximately 60 amino acids in length and is responsible for binding to the W-box of target gene promoter. The functional characterization of WRKYs indicates that they are widely involved in abiotic stress responses, such as heat stress [24] and cold stress [25]. The majority of genes associated with drought stress are regulated by ABA, and WRKY TFs play a pivotal role in ABA signaling pathway [26]. LtWRKY21 in the *Larrea tridentata* was shown to be induced by drought stress and to subsequently activate the ABA signaling pathway [27]. The high expression of *AtWRKY57* resulted in elevated ABA levels and enhanced drought tolerance of plants [28]. Additionally, WRKY TFs have been demonstrated to directly regulate ABA biosynthesis in other species, including bananas (*Musa acuminate*) [29] and sweet potatoes (*Ipomoea batatas*) [30]. Based on the above, it is likely that the WRKY TF family is involved in the regulation of norisoprenoid biosynthesis.

In 2014, three research teams identified a total of 59 WRKY TFs from ‘Pinot Noir’ grape. The functions of some WRKYs have been confirmed to be related with cold, salt or drought stresses [31–33]. In our previous study, WRKYs with the potential to regulate norisoprenoid biosynthesis were identified from grape berries by a yeast one-hybrid (Y1H) screening. VviWRKY70 has been confirmed to inhibit norisoprenoid accumulation by negatively regulating carotenoid production [22]. The present study focused on another group I WRKY TF, VviWRKY24, which was identified as an activator of *β*-damascenone biosynthesis by targeting *VviNCED1* expression and increasing endogenous ABA content. The objective of this study was to elucidate the relationship among the WRKY TF, ABA, and *β*-damascenone accumulation in grapes.

## Results

### Molecular characterization of VviWRKY24

**Figure 1:**
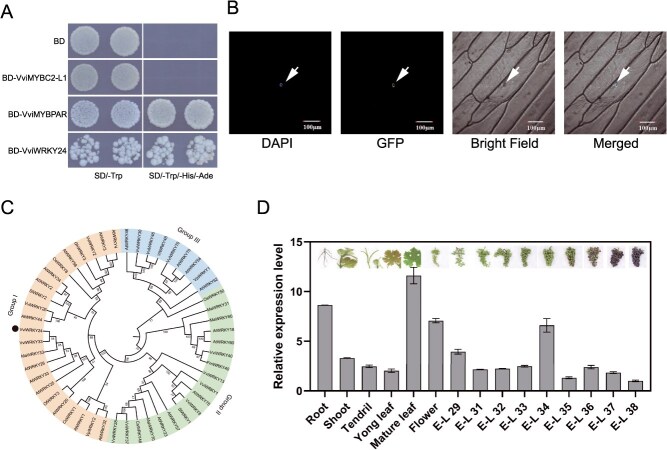
Molecular characterization and expression pattern of VviWRKY24. (A) Transcription activation activity assay. BD, pGBKT7 was used as a blank control; BD-VviMYBC2-L1 was used as a negative control; BD-VviMYBPAR was used as a positive control; SD/-Trp, SD medium lacking tryptophan; SD/-Trp/-His/-Ade, SD medium lacking tryptophan, histidine, and adenine. (B) Subcellular localization of VviWRKY24, DAPI (4′,6-diamidino-2-phenylindole) was used as a nuclear marker. (C) Phylogenetic tree of VviWRKY24. The black dot marks VviWRKY24. Accession numbers of the proteins are listed in Supplemental Table S1. (D) Spatiotemporal expression spectrum of *VviWRKY24*. The terms of E-L represented different stages of grape berry development [35]

In a previous study, seven WRKY TFs were identified in grape berries, and they may be involved in the regulation of norisoprenoid biosynthesis [22]. Subsequently, an additional transcription activation activity analysis was performed in yeast (*Saccharomyces cerevisiae*). As illustrated in Fig. 1A, the yeast cells that had been transformed with the BD-VviWRKY24 fusion protein were capable of growth on synthetic dropout (SD) medium lacking tryptophan, histidine, and adenine. This result suggested that VviWRKY24 (VIT_206s0004g07500) possessed a transcription-activation domain and it may function as a transcriptional activator. *VviWRKY24* was 1812 bp in length and encoded a protein with a molecular weight of 66.3 kDa. As is the case with the majority of TFs, VviWRKY24 was observed to localize in the nucleus (Fig. 1B). A phylogenetic tree was constructed with some WRKY TFs that had been validated functionally. VviWRKY24 shared high amino acid identity with OfWRKY3 (*Osmanthus fragrans*) and MaWRKY33 (*Musa acuminate*), which have been previously demonstrated to be associated with *β*-ionone biosynthesis in sweet osmanthus (*Osmanthus fragrans*) [16] and ABA biosynthesis in banana [29], respectively (Fig. 1C and Supplemental Table S1). All of the aforementioned proteins exhibited the characteristic two WRKY domains and two zinc finger motifs (Supplemental Fig. S1), which were the characteristic of group I WRKY subfamily [34].

### Expression patterns of VviWRKY24

The spatiotemporal expression spectrum revealed that *VviWRKY24* is widely expressed in various tissues of the grapevine, particularly in roots, mature leaves, and flowers, where it is highly expressed. In developing grape berries, the expression levels of *VviWRKY24* exhibited two minor peaks, one being in young berries at the E-L 29 stage and the other prior to véraison at the E-L 34 stage (Fig. 1D). At E-L 29, grape berries begin to enlarge, and at E-L 34, grape berries commence softening and sugar levels begin to increase [35]. The elevated expression levels of *VviWRKY24* at these two pivotal stages suggest that it may perform a crucial function in berry development.

To understand the characteristics of VviWRKY24 in response to environment factors, a series of treatments were performed on tissue culture grape plantlets. Given the high expression levels of *VviWRKY24* in leaves and roots (Fig. 1D), it can be speculated that VviWRKY24 may play a role in drought response processes. To simulate drought stress, PEG6000 was employed, and the resulting leaves and roots were subsequently collected for analysis. Following a 24-h treatment period, the leaves exhibited a loss of luster and wrinkling (Supplemental Fig. S2A). The expressions of *VviWRKY24* in leaves exhibited a fluctuation at 9 h or 12 h post-treatment, with no significant difference observed at 24 h (Supplemental Fig. S2B). The accumulation of ABA in the roots was markedly induced by drought (Supplemental Fig. S2A). Additionally, the gene expressions in roots were analyzed (Supplemental Fig. S3). The expression of *VviWRKY24* exhibited a pronounced response to drought, with a rapid increase (Supplemental Fig. S2A). Following a 24-h period of dark-pretreatment, the plantlets were subsequently exposed to light. elongated hypocotyl 5 (VviHY5) was employed as a positive control [36, 37], exhibiting a marked induction by light (Supplemental Fig. S2A). Conversely, *VviWRKY24* expression was persistently repressed by light (Supplemental Fig. S2A). With regard to temperature responsiveness, the expressions of *VviWRKY24* were rapidly repressed by elevated temperature but markedly enhanced to 3.4-fold following a 9-h cold treatment (Supplemental Fig. S2G and H). The observed sensitivity of *VviWRKY24* to environmental changes indicates the potential involvement of this gene in stress response and signal transduction processes.

### Transient expression of *VviWRKY24* positively regulates norisoprenoid biosynthesis in grape leaves and berries

**Figure 2:**
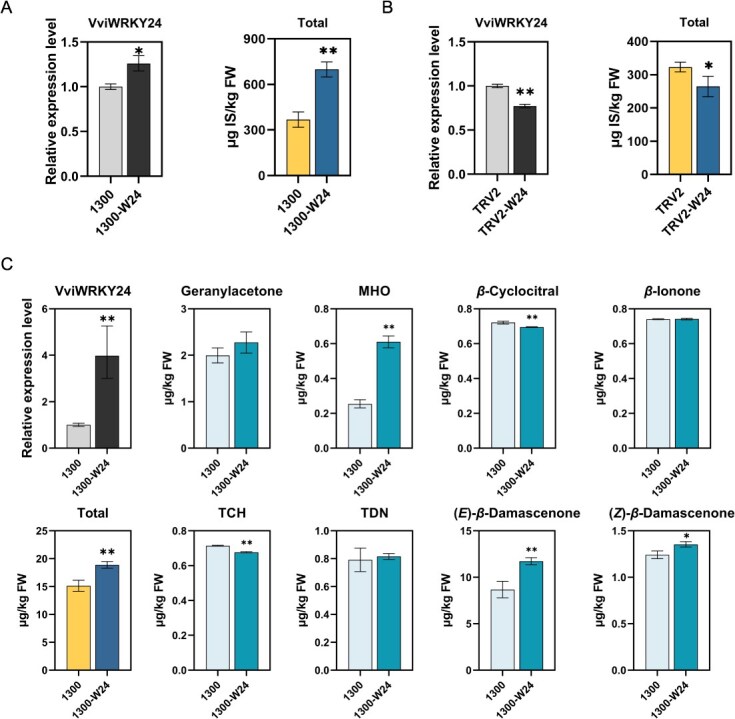
The effects of *VviWRKY24* transient expression on norisoprenoids. Expression levels of *VviWRKY24* and norisoprenoid accumulation in OE grape leaves (A) and VIGS grape leaves (B). The contents were determined by internal standard method (μg IS/kg FW). IS was 4-methyl-2-pentanol. (C) The norisoprenoid accumulation in OE grape berries. Error bars indicate means ± standard deviations (SD) from three replicates. Asterisks indicate significant differences relative to the empty vector group pCAMBIA1300 or pTRV2 (1300/TRV2) by *t*-tests (^*^, *P* < 0.05; ^**^, *P* < 0.01).

*VviWRKY24* was transiently expressed in grape leaves (*V. quinquangularis*) and grape berries (*V. vinifera*), respectively. Norisoprenoids were examined using gas chromatography–mass spectrometry (GC–MS), and five norisoprenoid compounds were detected in leaves of *V. quinquangularis* (Supplemental Fig. S4). The total norisoprenoid content was found to be elevated in *VviWRKY24*-overexpressing (OE) leaves in comparison to the control infiltrated with an empty vector (Fig. 2A). Conversely, the application of virus-induced gene silencing (VIGS) resulted in a notable reduction in the expression of *VviWRKY24*, accompanied by a considerable decline in norisoprenoid content (Fig. 2B).

Due to the almost undetectable presence of *β*-damascenone in leaves of *V. quinquangularis*, grape berries were used for further experiments. The expression level of *VviWRKY24* in transiently OE grape berries was found to be nearly fourfold higher than observed in the control group (Fig. 2C). Eight norisoprenoid compounds derived from different carotenoid precursors were identified in grape berries, including geranylacetone, MHO, *β*-cyclocitral, *β*-ionone, TDN, 2,2,6-trimethylcyclohexanone (TCH), and (*E*/*Z*)-*β*-damascenone. It was found that the OE berries exhibited elevated levels of MHO and (*E/Z*)-*β*-damascenone. In contrast, geranylacetone did not exhibit a significant increased, while *β*-cyclocitral and TCH demonstrated a slight decrease. While some differences were observed in the effects on various norisoprenoid compounds, the total norisoprenoid content in OE berries remained significantly higher than that in the control, due to (*E*)-*β*-damascenone representing the primary component of norisoprenoids in grape berries. These findings validate the hypothesis that VviWRKY24 positively regulate *β*-damascenone accumulation in grape berries.

### *VviWRKY24* overexpression facilitates *β*-damascenone synthesis in grape calli

**Figure 3:**
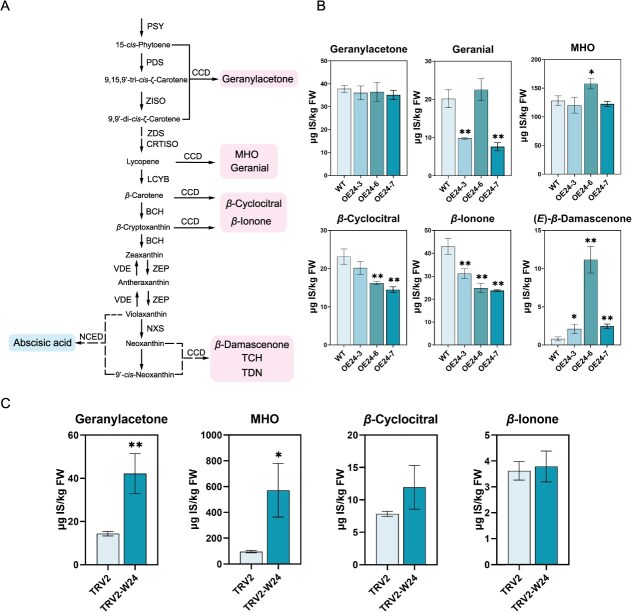
Effects of *VviWRKY24* expression on norisoprenoids in grape calli. (A) Schematic of norisoprenoid biosynthesis pathway. Solid arrows indicate direct reaction and dashed arrows indicate multiple-step reaction in the pathway. (B and C) Norisoprenoid accumulation in overexpressing (B) and VIGS (C) grape calli. The expression levels of *VviWRKY24* were shown in Fig. 4B. The contents were determined by internal standard method (μg IS/kg FW). IS was [D3]-linalool. Error bars indicate means ± SD from three replicates. Asterisks indicate significant differences relative to the WT or the empty vector group (TRV2) by *t*-tests (^*^, *P* < 0.05; ^**^, *P* < 0.01).

A stable genetic transformation was conducted in grape calli for the purpose of conducting further functional analysis of VviWRKY24. Three lines in which the gene was overexpressed (OE24–3, OE24–6, and OE24–7) were obtained (Supplemental Fig. S5). Six norisoprenoid compounds were identified in the OE lines, exhibiting disparate effects (Fig. 3A and B). The levels of geranylacetone, geranial, and MHO, which were derived from linear carotenoids at the upstream pathway, exhibited disparate trends in the three independent OE lines. The geranylacetone contents in three OE lines were not significantly different from those in the wild type (WT), while two of lines exhibited a decrease in geranial levels and only one line demonstrated a notable increase in MHO content. The levels of *β*-cyclocitral and *β*-ionone, which are produced directly from *β*-carotene, were significantly lower in the OE lines than that in the WT. In contrast, the level of *β*-damascenone, a compound produced at the downstream of carotenoid metabolic pathway, were significantly elevated in three OE lines. These findings suggest that overexpression of *VviWRKY24* in grape calli may promote the metabolic flow towards *β*-damascenone synthesis in the carotenoid downstream pathway.

A transient VIGS line (TRV-W24) was employed as an auxiliary reference. In order to reduce oxidative damage and enhance transformation efficiency, the VIGS grape calli were cultured in a dark environment and only four norisoprenoids were identified. It was observed that geranylacetone and MHO contents significantly increased when the expression of *VviWRKY24* was silenced, while the contents of *β*-cyclocitral and *β*-ionone were not significantly altered. This indicates that a decrease in *VviWRKY24* expression was more conductive to the synthesis of norisoprenoid compound at the upstream metabolic pathway (Fig. 3A and C). Based on the results of overexpression and silencing expression, it is hypothesized that VviWRKY24 affects the metabolic flux distribution and compositional proportion of norisoprenoid compounds in grape calli.

### VviWRKY24 has little effect on carotenoid accumulation

To ascertain whether the promotion of VviWRKY24 on the accumulation of norisoprenoids is achieved through an increase in the level of carotenoids, an assessment was conducted on the contents of carotenoids in the stable OE grape calli and in the transient OE grape berries. The data obtained are presented in Supplemental Fig. S6. In the three OE lines, with the exception for an increase in *β*-carotene content, *VviWRKY24* overexpression did not cause consistent alterations in the levels of other carotenoid compounds when compared with the WT, particularly neoxanthin, the precursor of *β*-damascenone. Similarly, no significant difference in carotenoid contents was observed in transient OE grape berries (Supplemental Table S2). These findings suggest that VviWRKY24 may directly modulate the metabolic pathway from carotenoids to norisoprenoids rather than the precursor contents.

### VviWRKY24 upregulates the expressions of genes involved in norisoprenoid biosynthesis

**Figure 4:**
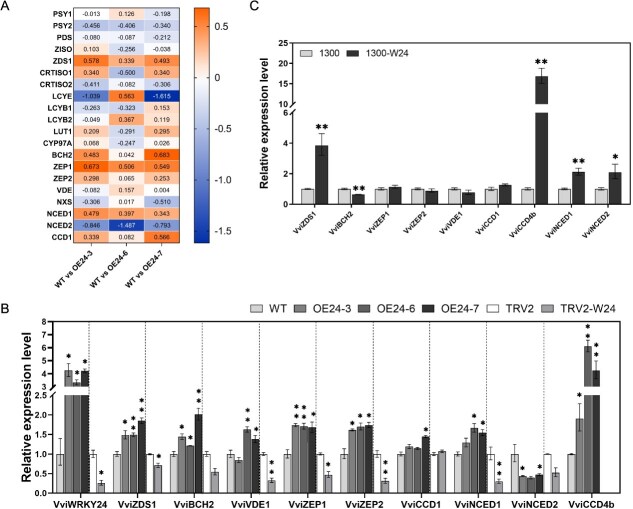
Effects of VviWRKY24 on the expressions of norisoprenoid biosynthesis-related genes. (A) DEGs involved in norisoprenoid biosynthesis in the *VviWRKY24* OE grape calli based on transcriptome analysis. WT was used as the control. Numbers in the boxes represent the intensity of log_2_ Fold change. (B) Gene expression levels in transgenic grape calli based on RT-qPCR. The expression level of *VviCCD4b* was not compared due to being too low in TRV-W24 line. (C) Gene expression levels in OE grape berry based on RT-qPCR. Error bars indicate means ± SEM from three replicates. Asterisks indicate significant differences relative to the WT or the empty vector group (WT/TRV2/1300) by multiple *t*-tests (^*^, *P* < 0.05; ^**^, *P* < 0.01).

A transcriptome analysis was conducted on OE grape calli (OE24–3, OE24–6, and OE24–7) with the objective of elucidating the regulatory mechanism of VviWRKY24 in norisoprenoid biosynthesis. The WT was served as the control. Gene transcript abundance was evaluated using the fragments per kilobase of transcript per million fragments mapped (FPKM). The log_2_ fold change was employed to quantify the difference in gene expression between the overexpressing grape calli and the WT (Supplemental Table S3). It was observed that the transcription levels of *VviPSY2* and *VviNCED2* were downregulated in all three OE lines, while the transcription levels of *ζ*-*carotene desaturase 1* (*VviZDS1*), *zeaxanthin epoxidase 1* (*VviZEP1*), and *VviNCED1* were upregulated (Fig. 4A). Given the potential function of VviWRKY24 as a transcription activator (Fig. 1A), it was postulated that these upregulated genes could be the candidate target genes of VviWRKY24.

To further validate the results, reverse transcription quantitative PCR (RT-qPCR) was performed on the OE lines and the VIGS line of grape calli to confirm the gene expression levels. The expression levels of *VviZDS1*, *VviZEP1*, and *VviNCED1* were found to be significantly elevated in the OE lines, while exhibiting a decline in the VIGS calli (Fig. 4B), which was in accordance with transcriptome analysis results. Additionally, in the grape berries that had been transiently overexpressed *VviWRKY24*, the expressions of *VviZDS1* and *VviNCED1* were also increased. However, *VviZEP1* expression exhibited no discernible difference, which was not in accordance with the observations made in grape calli. It was worth noting that although the transcription level of *VviCCD4b* in grape calli was too low to be detected in transcriptome analysis, RT-qPCR analysis indicated that this gene exhibited a markedly elevated expression in the OE calli lines and OE berries. In the OE grape berries, the expression of *VviCCD4b* was significantly upregulated, reaching a level nearly 20 times higher than that observed in the control (Fig. 4C). Based on the above results, *VviZDS1*, *VviNCED1,* and *VviCCD4b* were identified as candidate target genes of VviWRKY24.

### VviWRKY24 directly targets *VviNCED1* promoter to promote ABA biosynthesis

**Figure 5:**
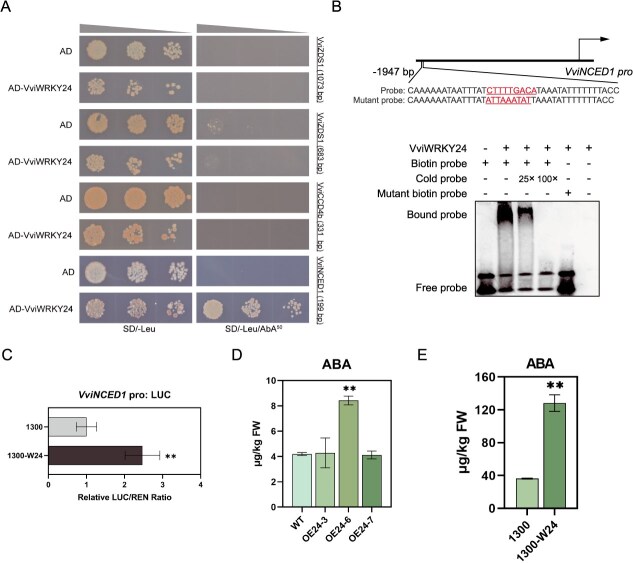
VviWRKY24 directly modulates ABA biosynthesis. (A) Y1H assay showed the interaction between VviWRKY24 and the promoters. AD, pGADT7 empty vector; AD-VviWRKY24, prey vector; SD/-Leu, SD medium lacking leucine; SD/-Leu/AbA, SD/-Leu supplemented with aureobasidin A (AbA, ng/ml). (B) The EMSA showed the binding of VviWRKY24 to the W-box in the *VviNCED1* promoter. (C) Dual-LUC assay demonstrated that VviWRKY24 activated the *VviNCED1* promoter. Error bars indicate means ± SD from six replicates. (D and E) ABA contents in *VviWRKY24* OE grape calli (D) and berries (E). Error bars indicate means ± SD from three replicates. Asterisks indicate significant differences relative to WT or the empty vector group (WT/1300) by *t*-test (^**^, *P* < 0.01).

The promoter regions of *VviZDS1*, *VviNCED1*, and *VviCCD4b* all contained at least one W-box. Based on these possible binding sites, the promoter fragments containing W-boxes were designed as the baits for one-on-one Y1H with VviWRKY24. The results demonstrated that only the yeast cells containing the VviWRKY24 and *VviNCED1* promoter fragment exhibited survival on the selective medium (Fig. 5A). It can be inferred that VviWRKY24 directly bond to *VviNCED1* promoter, and that no direct interaction occurs between VviWRKY24 and the promoter of *VviZDS1* or *VviCCD4b*.

To validate the Y1H outcome, an electrophoretic mobility shift assay (EMSA) was conducted. The biotin-labeled probe was designed to encompass the W-box within the *VviNCED1* promoter fragment. An excess of unlabeled probe with the identical sequence was employed as a competitive control. The labeled probe formed a blocking band with VviWRKY24 protein (bound probe). Upon the addition of an unlabeled probe, the intensity of the blocking band diminished and ultimately disappeared as the quantity of the unlabeled probe increased. This suggests that unlabeled probe competed with the labeled probe for interaction with the VviWRKY24 protein. Furthermore, when the W-box sequence in the probe was mutated, no bound probe was observed, thereby indicating that the W-box in the *VviNCED1* promoter is the binding site of VviWRKY24 (Fig. 5B).

A dual-luciferase assay (dual-LUC) was performed to elucidate the regulatory impact of VviWRKY24 on *VviNCED1* promoter activity. VviWRKY24 was observed to enhance the activity of the *VviNCED1* promoter by more than twice and to increase the expression of the reporter gene (Fig. 5C). These findings indicate that VviWRKY24 upregulates the expression of *VviNCED1* by directly binding to the W-box in the *VviNCED1* promoter.

Although NCED enzymes exhibit comparable cleavage dioxygenase activity to the CCDs, their cleavage sites and substrate specificity diverge [4, 38]. To date, no evidence has been indicated that VviNCED1 can directly cleavage carotenoids to produce *β*-damascenone. NCED1 is frequently regarded as a key enzyme in the biosynthesis of ABA [7]. It was therefore speculated that VviWRKY24 may regulate *β*-damascenone by increasing the level of ABA. To verify this hypothesis, the ABA content in the OE calli lines and OE grape berries was quantified. Although only OE24-6 line with the highest *VviNCED1* expression in OE calli showed a significant increase in ABA content (Fig. 5D), the overexpression of *VviWRKY24* in berries led to a notable elevation in ABA content, reaching a level that was 3.5 times higher than that observed in the control (Fig. 5E). These evidences support the hypothesis that the upregulation of *VviNCED1* expression by VviWRKY24 promotes the biosynthesis of ABA.

### VviWRKY24 activates ABA-induced *β*-damascenone biosynthesis

**Figure 6:**
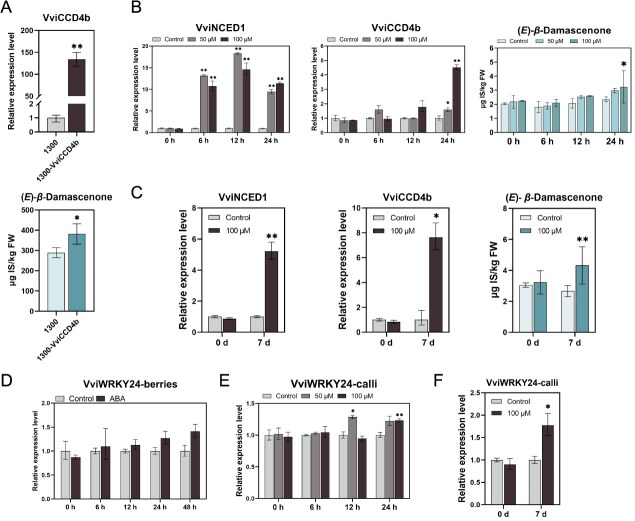
Effects of ABA on *β*-damascenone biosynthesis. (A) *β*-damascenone contents in *VviCCD4b* overexpressing grape leaves of *V. vinifera* L. cv Marselan and the empty vector control (1300). The contents were determined by internal standard method (μg IS/kg FW). IS was 4-methyl-2-pentanol. (B-F) The effects of ABA treatments on gene expressions and *β*-damascenone contents in WT calli (B, C, E, and F) and grape berries (D). The *β*-damascenone contents were determined by internal standard method (μg IS/kg FW). IS was [D3]-linalool. Error bars indicate means ± SD from three replicates. Asterisks indicate significant differences by multiple *t*-test (^*^, *P* < 0.05, ^**^, *P* < 0.01).

In OE24-6, along with a significant increase of ABA content, the expression of *VviCCD4b* as well as the content of *β*-damascenone were also at the highest levels (Figs 3B and 4B). Therefore, it was speculated that ABA induced *β*-damascenone accumulation by activating the expression of *VviCCD4b*. Our previous studies have demonstrated that exogenous ABA treatment of grape berries, can rapidly increase the expression of *VviNCED1*, *VviCCD4b*, and the content of *β*-damascenone [13, 15]. The cleavage dioxygenase activity of VviCCD4b has been characterized *in vitro* [38]. In this study, we first validated that overexpression of *VviCCD4b* increased the content of *β*-damascenone in the leaves of *V. vinifera* L. cv Marselan (Fig. 6A). Subsequently, we further investigated the effects of ABA on related gene expressions and *β*-damascenone contents in grape calli. Similar to the results in berries, *VviNCED1* exhibited sensitivity to ABA, with the expression levels rapidly increased from 6 to 24 h after ABA treatment. Furthermore, *VviCCD4b* expression also increased 24 h after treatment, slightly later than *VviNCED1* (Fig. 6B). Correspondingly, the content of *β*-damascenone began to accumulate at 24 h (Fig. 6B). Then we did a longer ABA treatment and got more pronounced results. The content of *β*-damascenone increased significantly with the up-regulation of *VviNCED1* and *VviCCD4b* (Fig. 6C). These results indicated that VviWRKY24 could regulate the synthesis of *β*-damascenone by directly promoting the expression of *VviNCED1*, activating ABA biosynthesis and inducing the expression of *VviCCD4b*.

The expression of *VviWRKY24* was also examined. No notable alterations (less than 1.5 times) were discerned in either grape calli or grape berries following short period of ABA treatment, suggesting that *VviWRKY24* expression itself was not stimulated directly by ABA. The increase in *VviWRKY24* expression after 7 days of ABA treatment may be the result of other feedback regulation (Fig. 6D-F).

## Discussion

**Figure 7:**
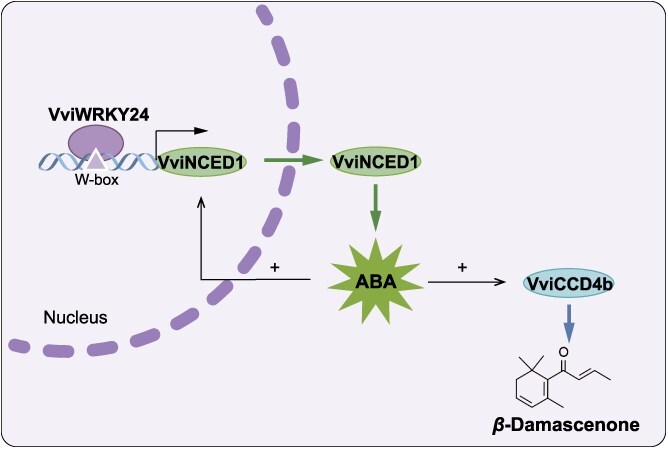
A model for illustrating the regulation of VviWRKY24 on ABA-induced *β*-damascenone biosynthesis in grape berries based on the findings of this study. VviWRKY24 activates ABA biosynthesis by binding the W-box in the *VviNCED1* promoter. ABA induces *β*-damascenone accumulation by upregulating the expression level of *VviCCD4b* and by feedback upregulating *VviNCED1* expression.

*β*-Damascenone represents a significant indicator for the assessment of wine aroma quality, with a notable impact on the floral and fruity aroma profiles observed in wines produced from non-aromatic (also called as neutral type) grape varieties, including *V. vinifera* L. cv Cabernet Sauvignon and Merlot, which are the most extensively produced red wine varieties in China. In our previous study, two repressors were demonstrated to negatively regulate the biosynthesis of norisoprenoids in grape [21, 22]. The present study identified a positive regulatory factor of *β*-damascenone biosynthesis, VviWRKY24, in grape berries. A model is proposed for elucidating the regulatory mechanism by which VviWRKY24 promotes *β*-damascenone synthesis by targeting *VviNCED1* to increase ABA levels in grapes. VviWRKY24 directly activates the expression of *VviNCED1*, thereby promoting ABA synthesis. ABA upregulates the expressions of *VviCCD4b*, which leads to an increase in *β*-damascenone contents (Fig. 7).

### VviWRKY24 exerts disparate regulatory effects on the synthesis of diverse norisoprenoid compounds

The generation of norisoprenoids is accompanied by the cleavage of diverse carotenoid compounds. In accordance with the up-to-down chemical alterations observed in the carotenoid biosynthetic pathway, norisoprenoid compounds are produced from carotenoids in a sequential manner, namely geranylacetone, MHO, *β*-cyclocitral, *β*-ionone, and *β*-damascenone [39, 40]. The results of this study on grape leaves, calli, and berries indicate that the elevated expression of *VviWRKY24* is conducive to the accumulation of downstream norisoprenoid compounds. However, slight variations were observed in the regulatory effects on grape calli, where the overexpression of *VviWRKY24* resulted in an increase in *β*-damascenone content, while the levels of upstream norisoprenoid compounds exhibited a decrease or no significant difference. Conversely, the silencing of *VviWRKY24* expression in grape calli led to an increase in the levels of upstream norisoprenoid compounds (Fig. 3). It was proposed that the reduction in *VviWRKY24* expression in grape calli might result in an increase in the availability of upstream precursors, thereby enhancing the production of upstream norisoprenoid compounds.

The carotenoid pathway has been demonstrated to possess a strong feedback mechanism, exhibiting tissue-specific effects [41–43]. A study in tomatoes has revealed that the elevated expression levels of *phytoene desaturase* (PDS) in all aerial tissues can result in an altered accumulation of carotenoids in a tissue-specific manner, indicating a unique pattern of carotenoid self-regulation [44]. These previous findings may provide an explanation for the influence of altered carotenoid metabolic flux on the production of upstream or downstream norisoprenoid compounds.

Overall, this study confirms that elevated expression of *VviWRKY24* significantly enhances the metabolic flux towards the synthesis of the downstream compound *β*-damascenone. It is noteworthy that *β*-damascenone represents the highest concentration of norisoprenoids in grape berries [3]. The synthesis of *β*-damascenone was markedly enhanced by VviWRKY24 in transiently overexpressed grape berries, thereby providing compelling evidence that VviWRKY24, as a positive regulatory factor, plays a pivotal role in the biosynthesis of *β*-damascenone in grape berries.

### VviWRKY24 targets *VviNCED1* to elevate ABA-induced *β*-damascenone

The biosynthesis of *β*-damascenone involves multiple reaction steps, and the key enzymes involved in the enzymatic reactions in grapes are not entirely clear [2, 40, 45]. Our previous research has indicated that ABA treatment on grape berries can elevate the endogenous ABA and influence the accumulation of *β*-damascenone [12]. Further studies have demonstrated that ABA treatment of grape berries can rapidly increase the expression of *VviNCED1*, *VviCCD4b*, and the content of *β*-damascenone [13, 15]. In this study, similar results were confirmed in grape calli (Fig. 6). *VviCCD4b* expression was significantly correlated with the content of *β*-damascenone during grape ripening [21]. In this study, we confirmed that overexpression of *VviCCD4b* increased the content of *β*-damascenone (Fig. 6A), indicating that VviCCD4b was a key enzyme in *β*-damascenone synthesis. This study demonstrates that VviWRKY24 is involved in ABA biosynthesis by directly upregulating the expression of *VviNCED1*, which in turn exerts a regulatory effect on *β*-damascenone synthesis mediated by VviCCD4b.

WRKY TFs have been demonstrated to play a pivotal role in the biosynthesis and signaling of ABA [26]. Research indicates that specific WRKY TFs are directly involved in regulating ABA biosynthesis, subsequently stimulating or suppressing downstream of ABA signaling, and ultimately eliciting plant physiological responses. For example, AtWRKY33 has been demonstrated to reduce ABA levels by directly downregulating the expressions of *AtNCED3* and *AtNCED5* [46]. Furthermore, OsWRKY50 has been demonstrated to regulate ABA-dependent seed germination and seedling growth by downregulating the expression of *OsNCED5* in *Oryza sativa* L. [47]. In grapes, VviWRKY31 has been observed to promote ABA biosynthesis, potentially by activating the expressions of *VviZEP1*, *VviABA2* (*xanthoxin dehydrogenase*), *VviNCED*, and *VviAAO* (*abscisic–aldehyde oxidase*) [48]. Similarly, our study indicates that VviWRKY24 directly interacts with the W-box on the *VviNCED1* promoter, upregulating its expression and resulting in an increase in ABA content (Fig. 5).

A feedback loop of ABA has been identified in plants. In other words, some WRKY TFs have been observed to respond to ABA induction, which in turn modulates ABA biosynthesis. One such example is that of VviWRKY37, which has been identified as an ABA-induced TF with a role in bud dormancy. This TF has been demonstrated to downregulate the expression of *VviHYD* (*ABA 8′ hydroxylase*), thereby inactivating ABA catabolism and promoting the accumulation of endogenous ABA [49]. In the study conducted by Wang L. et al [15], the expression of *VviWRKY24* (referred to as *VvWRKY28* in their study) in grape leaves was found to be induced by ABA. The induction occurred within an hour after ABA treatment, with a nearly doubled increase. Additionally, our previous research indicates that the expression of *VviNCED1* in grape berries is induced by ABA [15]. However, the present study shows that the expression of *VviWRKY24* is not significantly elevated within 48 h after ABA treatment in either grape calli or berries, exhibiting only a slight upward trend (less than 1.5 times) (Fig. 6). Therefore, we speculate that VviWRKY24 is not sensitive to ABA signaling, at least in the short term. Instead, this TF acts the upstream of ABA signal, directly regulating ABA biosynthesis and consequently promoting ABA-induced gene expression and metabolism.

### VviWRKY24 may be involved in other ABA-mediated physiological activities

The alterations in ABA levels regulate the growth of roots and shoots in response to both biotic and abiotic stresses [50]. It has been demonstrated that VviWRKY24 plays a role in the accumulation of proanthocyanidins and resistance to powdery mildew in grapevine [51]. The present study revealed that the expression levels of *VviWRKY24* were higher in roots and leaves than in berries (Fig. 1D). Furthermore, the expression of *VviWRKY24* in grapevine roots was significantly enhanced by drought induction, accompanied by a notable elevation in ABA content (Supplemental Fig. S2C and D). This suggests that VviWRKY24 may be involved in ABA-mediated drought tolerance regulation in grapevine roots. In *Arabidopsis thaliana*, AtWRKY57 has been demonstrated to directly bind to the W-box in the *AtNCED3* promoter, thereby enhancing plant drought tolerance by increasing ABA levels [28]. A comparable phenomenon was observed in banana, where MaWRKY80 was identified as a positive regulator of drought tolerance, upregulating *MaNCED* expression and increasing ABA content [52]. The ABA level is markedly elevated in response to water deficit in grapes [53]. VviNCED1 plays a pivotal role in the response to water deficit in grape leaves and roots, exhibiting elevated expression levels and ABA content in drought-tolerant grape varieties [54]. The current study demonstrated that the expression of *VviWRKY24* and the content of ABA were elevated in the roots subjected to drought stimulation, whereas the expression of *VviNCED1* remained unaltered and other norisoprenoid biosynthesis-related genes exhibited different responses (Supplemental Fig. S2). The results suggest that the existence of a complex mechanism involving VviWRKY24 in the ABA-mediated response to drought induction.

Numerous studies have established the role of ABA in the physiological response to low temperatures. A study on post-harvest grapes revealed that the ABA content in the peel of mature grapes is elevated following low-temperature treatment [55]. A recent study on tea plants has indicated that the glycosyltransferase UGT85A53 can integrate the regulatory mechanisms to enable tea plants to cope with low temperature and drought stress through ABA glycosylation [56]. With regard to the function of WRKY TFs in the response to low temperatures, studies in bananas [29] and tea plants [57] have demonstrated the involvement of WRKY TFs. Furthermore, our study demonstrates that the expression of *VviWRKY24* is significantly induced by low temperature (Supplemental Fig. S2H), which may consequently modulate the expression of ABA-related glycoside hydrolase and glycosyltransferase genes (Supplemental Table S3). It is important to note that while the current data indicate that *VviWRKY24* expression is induced following drought and low-temperature treatments, there is currently no evidence that this induction will promote ABA biosynthesis through *VviNCED1* expression upregulation and enhance the resistance. This will be determined in future research.

## Materials and methods

### Plant material and grape berry sampling

The grape materials used for spatiotemporal expression spectrum were identified as those employed in the preceding study [22]. The eight-week-old tissue culture grape plantlets used for expression pattern analysis under drought stress, light, or temperature treatments were *V. vinifera* L. cv Cabernet Sauvignon, cultivated on McCown woody plant medium with 30 g/L sucrose, 7 g/L agar and 2 mg/L indole-3-butyric acid at pH 5.9. The tissue culture grape plantlets and tobacco (*Nicotiana benthamiana*) plants were cultivated in a growth chamber at 25°C in 16-h/8-h light/dark cycle. The light intensity was 250 μmol photons m^−2^·s^−1^.

For transient transformation, the berries of *V. vinifera* L. cv Ruidu Kemei at E-L33 (hard berries before véraison) and the leaves of *V. quinquangularis* L. were collected from Institute of Forestry and Pomology, Beijing Academy of Agriculture and Forestry Sciences vineyard (39°58′N and 116°13′E, Beijing, China) in 2023. The leaves of *V. vinifera* L. cv Marselan were collected in 2024.

The ‘Cabernet Sauvignon’ calli was preserved through long-term subculture in our laboratory. The calli was cultured on B5 medium in the absence of light and subcultured at 25-day intervals, as previously reported [22].

The *V. vinifera* L. cv Jingxiangyu grape berries at E-L 33 used for ABA treatment were identical to those employed in our previous study [15], which were collected from the greenhouse of the Yanhuai Valley Grape and Wine Industry Federation in Yanqing (40°30′N，116°1′E, Beijing, China) in 2023.

### Treatment conditions

Light treatment experiment was performed on eight-week-old tissue culture grape plantlets of ‘Cabernet Sauvignon’. The plantlets were subjected to a 24-h-pretreatment in darkness, with the objective of eliminating the influence of photoperiod. Following this, they were exposed to light. Sampling was conducted at 0, 3, 6, 9, 12, and 24 h post-treatment. *VviHY5*, which has been confirmed as a light-responsive gene, was selected as the positive control [36, 37].

The plantlets subjected to temperature treatments were maintained in a stable darkness throughout the course of the experiment. The planets in the high-temperature groups were placed in an artificial climate chamber maintained at 40°C. Samples of their leaves were taken at 0, 3, 6, 12, and 24 h after treatment. The low temperature groups were placed in an artificial climate chamber maintained at 4°C and the leaves were sampled at 0, 3, 6, 9, 16, and 24 h after treatment. The control groups were maintained at 25°C. Each group comprised a minimum of nine leaves from three plantlets and was divided into three replicates.

A solution of 10% PEG6000 (M/V) was employed to simulate the effects of drought stress according to Yiyong Ma [58]. The roots of experimental group plantlets were immersed in sterilized 10% PEG6000 solution, while the roots of the control group were immersed in sterilized water. All groups were cultivated in the conventional growth chamber. Samples were collected at 0, 6, 9, 12, and 24 h after treatment. The leaves and roots of the tissue-culture grape plantlets were harvested for subsequent analysis. Each group comprised at least three plantlets, which were subdivided into three replicates.

The ABA immersion experiment was conducted on grape calli and ‘Jingxiangyu’ grape berries, respectively. As previously documented in our research [21], the calli was immersed in ABA solutions of 50 and 100 μM in water for a period of 15 min, after which it was transferred to a medium containing sterile filter paper for continue cultivation. Water served as the control. Samples were collected at 0, 6, 12, and 24 h post-treatment, with three replicates from each group. In accordance with the methodology outlined in our previous study [15], grape berries were soaked in solution containing 1000 mg/L ABA and 0.1% (V/V) Tween 80 for 15 min. A control solution of water with 0.1% (V/V) Tween 80 was prepared. Once the surface of berries has dried naturally, they were placed in a sealed culture dish with cling film. Samples were collected at 0, 6, 12, 24, and 48 h after treatment, with three replicates from each group. For long-term processing, according to previous report [21], the calli was transferred to the B5 medium containing 100 μM ABA，samples were collected after 7 days, with three replicates from each group.

### Gene cloning and sequence analysis

The *VviWRKY24* coding sequence (CDS) was cloned from the ‘Cabernet Sauvignon’ cDNA library using the primers shown in Supplemental Table S4. The amino acid sequences of the WRKY proteins were obtained from NCBI protein database (https://www.ncbi.nlm.nih.gov/protein) and the accession numbers are listed in Supplemental Table S1. A phylogenetic tree was constructed using MEGA 6 and iTOL (https://itol.embl.de/itol.cgi) with the neighbor-joining method. Sequence alignments were conducted using DNAMAN 8 (LynnonBiosoft, USA). The protein tertiary structure (ID: F6GUH8) was predicted by AlphaFold in the UniProt database (https://www.uniprot.org/).

### Subcellular localization

The VviWRKY24-green fluorescent protein (GFP) fusion protein was expressed using pEZS-NL vector for the purpose of conducting a subcellular localization analysis. The primers used are detailed in Supplemental Table S4. The plasmid was transient transformed into epidermal cells of onion (*Allium cepa*) using particle bombardment technology. Following a 24-h incubation, the GFP fluorescene signal and DAPI (4′,6-diamidino-2-phenylindole) signal were imaged using an Olympus FV1000 laser scanning confocal microscope (Tokyo, Japan). The wavelength parameters employed were identical to those utilized in our previous study [22].

### Transcriptional activity assay

The transcriptional activity of VviWRKY24 was examined through the use of the yeast self-activation system, specifically the AH109 strain [59]. The CDS of *VviWRKY24* was cloned into the pGBKT7 vector, and the recombinant plasmid was transformed into the AH109 yeast strain. Two TFs, VviMYBPAR [60] and VviMYBC2-L1 [61], which have been previously demonstrated to function, were employed as a positive control and a negative control, respectively. The transformed yeast solution was plated onto SD medium lacking tryptophan (SD/-Trp) and SD medium lacking tryptophan, histidine, and adenine (SD/-Trp/-His/-Ade) and was cultured for five days.

### Genetic transformation

Stable overexpression of *VviWRKY24* in grape calli was achieved through the use of the pCXSN-VviWRKY24-FLAG vector in *Agrobacterium tumefaciens* strain GV3101. The method of transformation was previously described in detailed in reference [15]. The overexpressing lines and WT line were cultured in a climate chamber with a 16-h/8-h light/dark photoperiod for seven days to facilitate the additional carotenoids and norisoprenoids prior to further analysis. Identification of transgenic calli at gDNA level was performed using the primers listed in Supplemental Table S4.

A transient overexpression of *VviWRKY24* was conducted using the pCAMBIA1300-VviWRKY24 vector in *A. tumefaciens* strain GV3101. VIGS was employed to achieve gene silencing through the pTRV1 and pTRV2 vectors. The recombined plasmids were transformed into *A. tumefaciens* strain GV3101. An equal amount of mixture containing pTRV1 and pTRV2 was used as the control group, while an equal amount of mixture containing pTRV1 and pTRV2-VviWRKY24 was employed as the experimental group. The methodologies employed for the *A. tumefaciens* infection method of grape leaves and berries have been previously described in detail [15].

Additionally, transient gene silencing in grape calli was conducted using the VIGS system, with the transformation process executed in accordance with the methodology employed for stable overexpression in grape calli. To mitigate the adverse effects of browning and enhanced the transient expression efficiency, the samples were taken directly following the dark cultivation period, instead of light treatment.

### Y1H analysis

Y1H analysis was performed in accordance with the Matchmaker Gold Y1H System (Clontech, USA). The genome was extracted from tissue culture grape plantlets of ‘Cabernet Sauvignon’ using the Plant Genome Extraction Kit (DP3112, Beijing Bioteke, China). The promoter fragments cloned from the genome were utilized as the baits, which were subsequently introduced into the pAbAi vectors. The CDS of *VviWRKY24* was introduced into the pGADT7 vector as the prey. The primers used are listed in Supplemental Table S4. The baits and prey were subsequently transformed into the Y1HGold yeast strain. The detailed procedures have been previously described [22].

### Dual-LUC

The promoter of *VviNCED1* (gene upstream 1947 bp) was isolated from ‘Cabernet Sauvignon’ cultivar and was subsequently incorporated into the pGreenII 0800 double-reporter vector. The *VviWRKY24* CDS was introduced into the pCAMBIA1300 vector to conduct the effector. An empty vector was set as the control. The dual-LUC assay was conducted in tobacco leaves in accordance with the previously described methodology [21]. The primers used are detailed in Supplemental Table S4.

### EMSA

The CDS of *VviWRKY24* was optimized for prokaryotic expression using the online tool https://www.novopro.cntoolscodon-optimization.html. Given the difficulty in expressing the entire protein, the conserved DNA binding domain (from the 763rd bp to the 1455th bp of CDS) of *VviWRKY24* was synthesized by Beijing Tsingke Biotech Co., Ltd. The binding domain was expressed using the pCzn1-His vector in Arctic Express strain (*Escherichia coli*). The promoter fragment (biotin probe) was synthesized and labeled with biotin by Sangon Biotech (Shanghai, China). A labeled probe with mutated oligonucleotides (marked in red letters) was synthesized as a mutant biotin probe. The unlabeled probe (cold probe) was used as the competitor. The EMSA was conducted following the previously described method [22].

### RNA extraction, cDNA synthesis, and RT-qPCR analysis

The total RNA was extracted using a Universal Plant Total RNA Extraction Kit (RP3302, Beijing BioTeke, China). cDNA was synthesized by HiScript II Q RT SuperMix for qPCR (R223, Vazyme, China) from 1 μg total RNA. RT-qPCR was performed using ChamQ Universal SYBR qPCR Master Mix (Q711, Vazyme, China), with the data acquisition performed on the CFX96 Real-Time System (Bio-Rad, USA). Quantification was achieved through the 2^-ΔΔCT^ method. The *UBIQUITIN* was used as the reference gene. The primers used are presented in Supplemental Table S4. Each reaction was conducted in triplicates, and the resulting data was analyzed using the CFX Maestro Software (BIO-RAD, USA).

### Transcriptome analysis by RNA-seq

Four groups were subjected to transcriptome analysis: WT calli in light and three independent *VviWRKY24* overexpressing calli lines in light (OE24–3, OE24–6, and OE24–7). The RNA sequencing libraries were constructed using the Biomarker Technologies platform (Beijing, China), and the sequencing was performed using an Illumina HiSeq platform. The raw data were mapped to the *V. vinifera* PN40024 genome 12X.2 and annotated in comparison with the V2.1 version. Gene transcription levels were evaluated by FPKM, and further data analysis was completed using BMKCloud (www.biocloud.net). Transcriptome data have been uploaded to the NCBI SRA database with the accession number PRJNA833286.

### Metabolite measurements

Sample preparations and norisoprenoid determinations were performed in accordance with a previously published study, with some modifications [22]. For grape calli, a solution was prepared by blending NaCl (1.5 g), citric acid/sodium citrate buffer (pH 3.0, 3 ml), sample powder (4.0 g), and [D3]-linalool as the internal standard (IS, 10 μL, 5 × 10^−3^ g/L). For leaves, a solution was prepared by blending NaCl (1.0 g), citric acid/sodium citrate buffer (pH 3.0, 5 mL), sample powder (1.0 g) and 4-methyl-2-pentanol (10 μL, .01 g/L, IS) in a sample vial. The mixture was used for GC–MS determination, with three replicates for each group of samples.

The berry samples were prepared as described previously [62]. The powder of grape berries (70 g) devoid of pedicels and seeds were blended with 1 g PVPP and 0.5 g *β*-D-glucolactone. Following a four-hour maceration at 4°C, samples were subjected to centrifugation, after which the resulting supernatant was collected. A solution was prepared by blending NaCl (1.0 g), sample supernatant (5 mL), and 4-methyl-2-pentanol (10 μL, 1 g/L, IS) in a sample vial for GC–MS determination, with three replicates for each group of samples. The norisoprenoids were concentrated by head-space solid-phase microextraction. The compounds were identified on the basis of obtained mass spectra and retention index (RI), compared with those of reference standards and compounds in NIST14 MS database. The norisoprenoid concentrations of berries were quantified using standard curves and were expressed as μg/kg fresh weight (FW). The concentrations in leaves and calli were quantified by internal standard method and were expressed as μg IS/kg FW.

The extraction and determination of carotenoid in grape calli were performed using an Agilent 1290 series UPLC in tandem with an Agilent 7670B QqQ-MS, as previously described [22]. The IS was 8′-apo-β-carotenal (20 μg/mL). The concentrations of carotenoids were quantified by means of standard curves and were expressed as μg/kg dry weight (DW). Each group of samples contained three replicates.

The extraction method of ABA from grape berries was identical to that previously described [15]. The ABA contents were determined by an Agilent 1290 series UPLC in tandem with Agilent 7670B QqQ-MS, in accordance with the previously published methodology [63]. Triphenylphosphate (TPP, 0.1 mg/L) was used as the IS and ABA contents were quantified through the standard curve and expressed as μg/kg FW. Each group of samples consisted of three replicates.

### Statistical analysis

The bar charts were constructed using GraphPad Prism version 9. All experiments were conducted in at least three parallelisms, and the resulting data were presented as means ± SD. Significant differences between means were defined by multiple *t*-tests (^*^, *P* < 0.05; ^**^, *P* < 0.01).

### Accession numbers

The gene and protein sequences mentioned in this study are available in the genome database of *V. vinifera*-PN40024 version 12x.V2 (http://www.grapegenomics.com/pages/PN40024/) or in the NCBI. Accession numbers and primers were shown in Supplemental Tables S1 and S4.

## Supplementary Material

Web_Material_uhaf017

## Data Availability

Raw data of RNA-seq were uploaded in the SRA database and the accession number is PRJNA833286. Other data used in this study are available from the corresponding authors upon reasonable request.
