# Supplementary material for: VviWRKY24 promotes β-damascenone biosynthesis by targeting VviNCED1 to increase abscisic acid in grape berries
Source: Hortic Res. 2025 Jan 15;12(5):uhaf017. doi: 10.1093/hr/uhaf017 (PMC11975394; doi:10.1093/hr/uhaf017)
Supplement: Web_Material_uhaf017 [file web_material_uhaf017.zip › Supplemental Figures.docx]

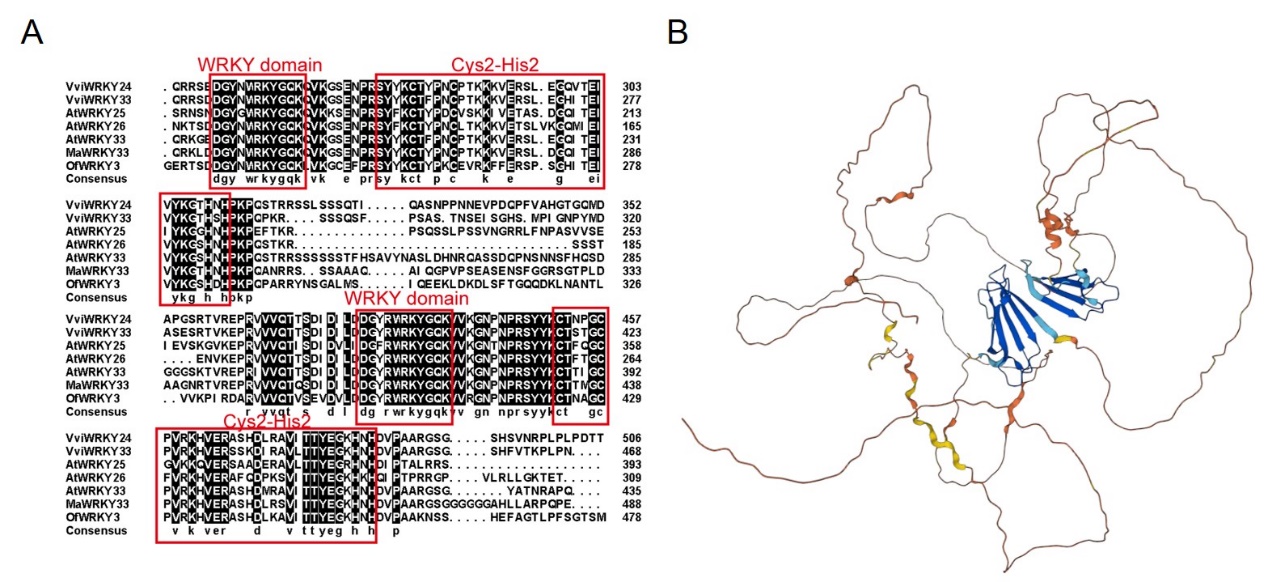


**Supplemental Figure S1.** Conserved domain analysis and 3D structure of VviWRKY24. The red lines in A. and blue structure in B. mark conserved domains.


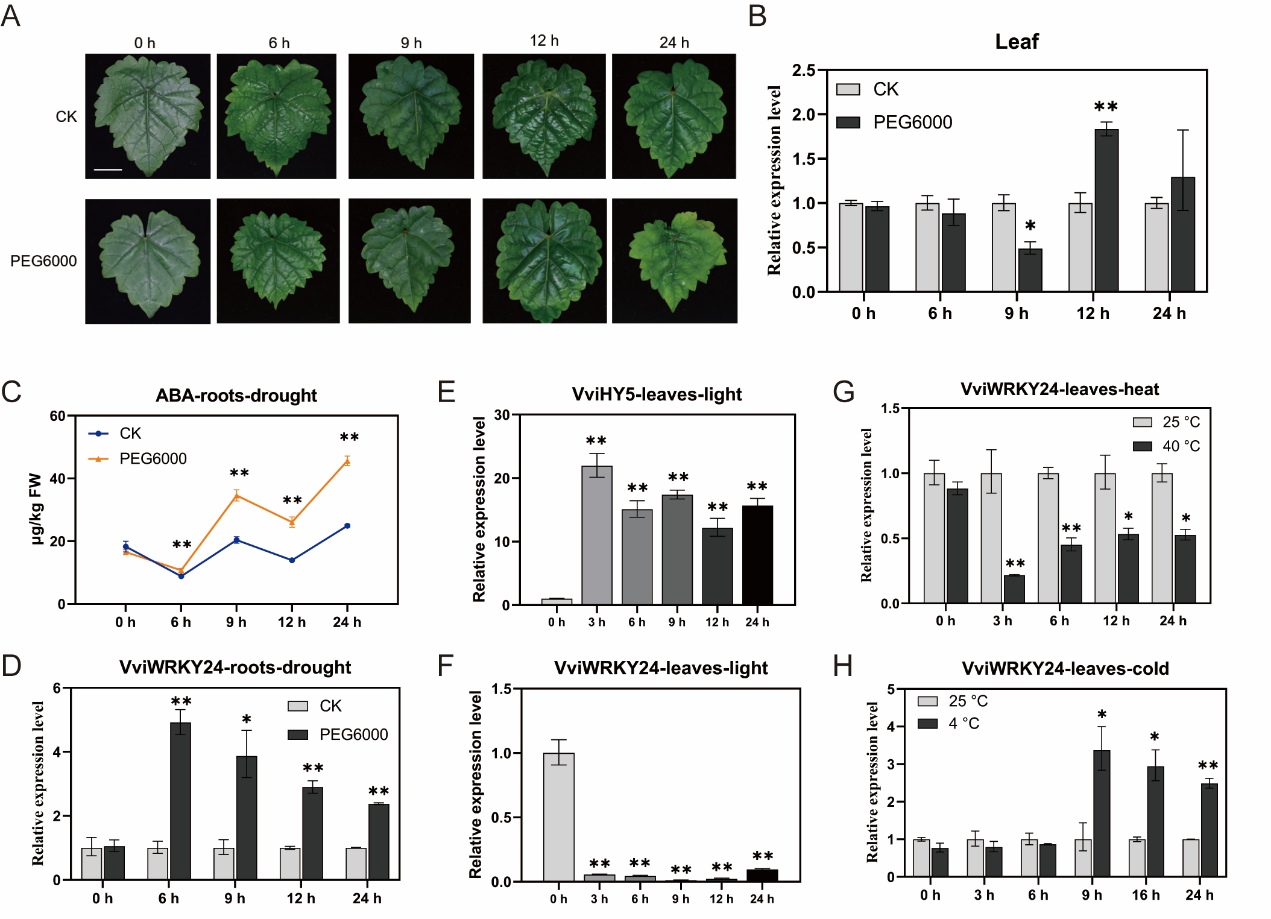


**Supplemental Figure S2.** The response of *VviWRKY24* to abiotic stresses. A. Phenotypes of leaves of tissue culture plantlets after PEG6000 treatment, the scale bar is 1 cm. B. Relative expression levels of *VviWRKY24* in leaves after PEG6000 treatment. C. ABA contents and D. gene expressions in roots after PEG6000 treatment. E―H. The gene expressions in leaves under light treatment (E and F), heat treatment (G) and cold treatment (H), respectively. Error bars indicate means ± SEM from three replicates. Asterisks indicate significant differences by *t*-tests (*, *p* < 0.05; **, *p* < 0.01).


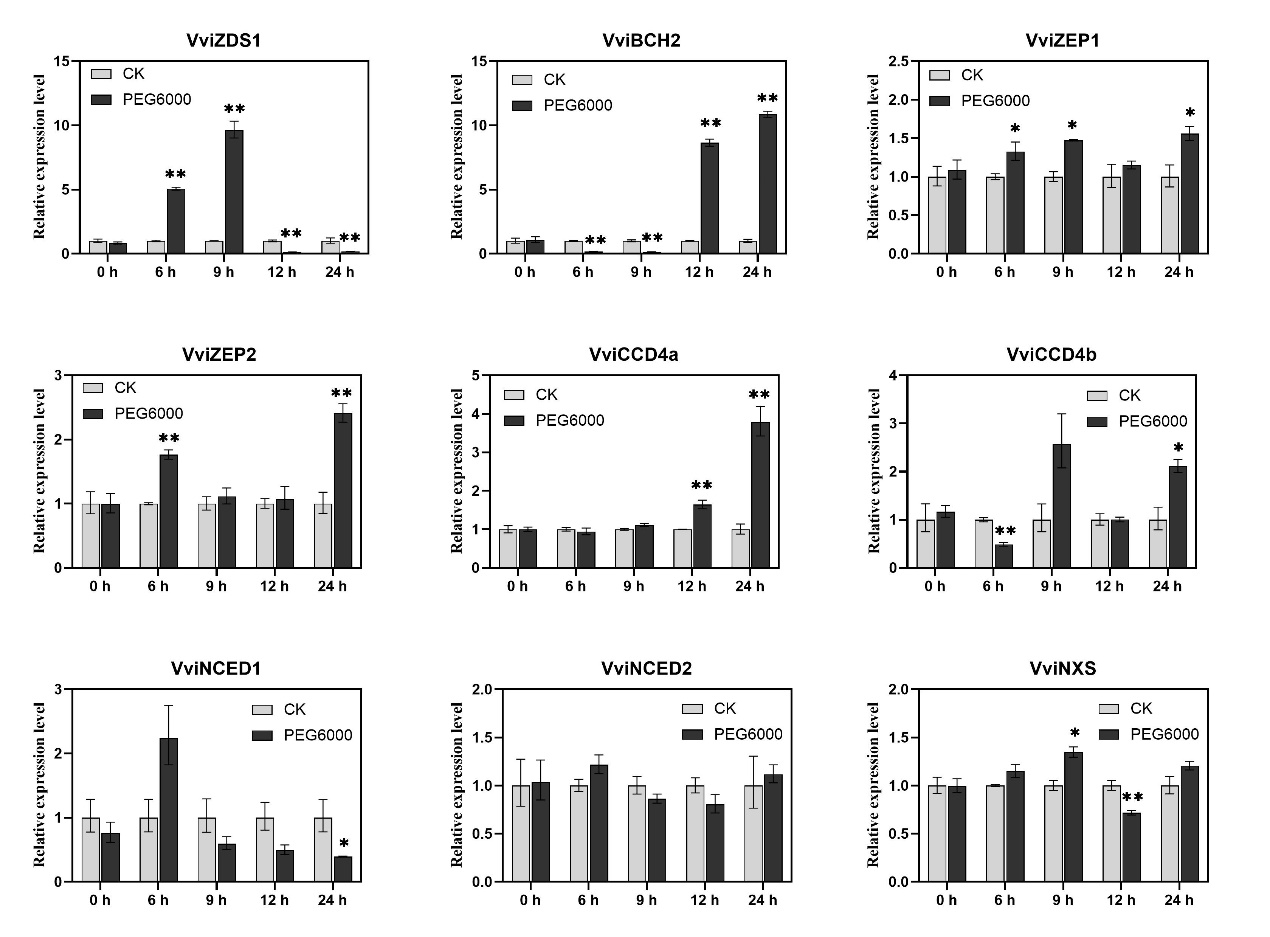


**Supplemental Figure S3.** The gene expressions in grape roots after PEG treatment. Relative gene expression levels in grape roots after PEG6000 treatment, error bars indicate means ± SEM from three replicates, water was used as controls, asterisks indicate significant differences by *t*-tests; *, *p* < 0.05; **, *p* < 0.01.


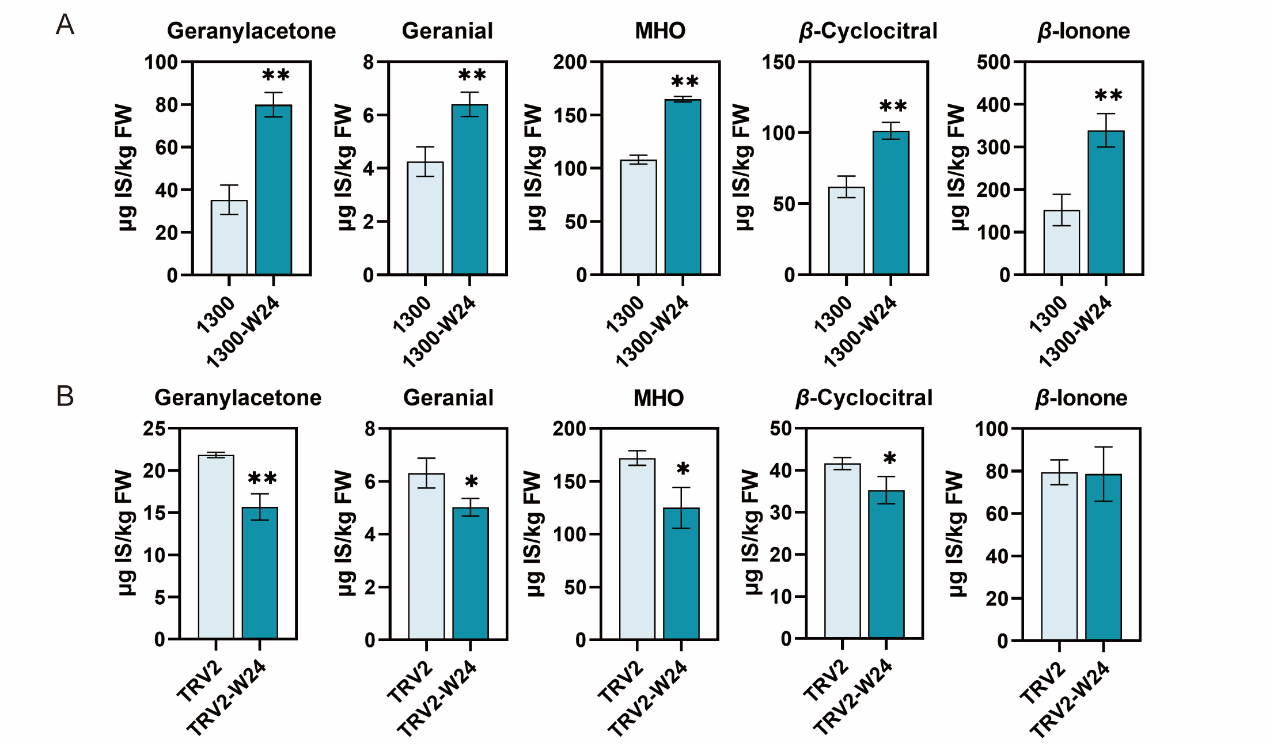


**Supplemental Figure S4.** The effects of *VviWRKY24* transient expression on norisoprenoids in *V. quinquangularis*. Norisoprenoid contents in A. overexpressing and B. VIGS leaves, the contents were determined by internal standard method (μg IS/kg FW), IS was 4-methyl-2-pentanol. Error bars indicate means ± SD from three replicates. Empty vector transformed leaves were used as controls; asterisks indicate significant differences by *t*-tests; *, *p* < 0.05; **, *p* < 0.01.


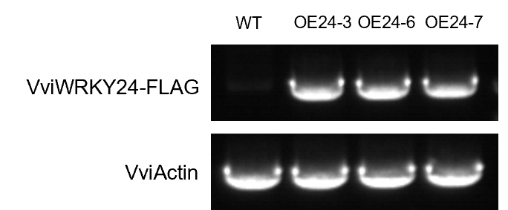


**Supplemental Figure S5.** Identification of transgenic calli at gDNA level.


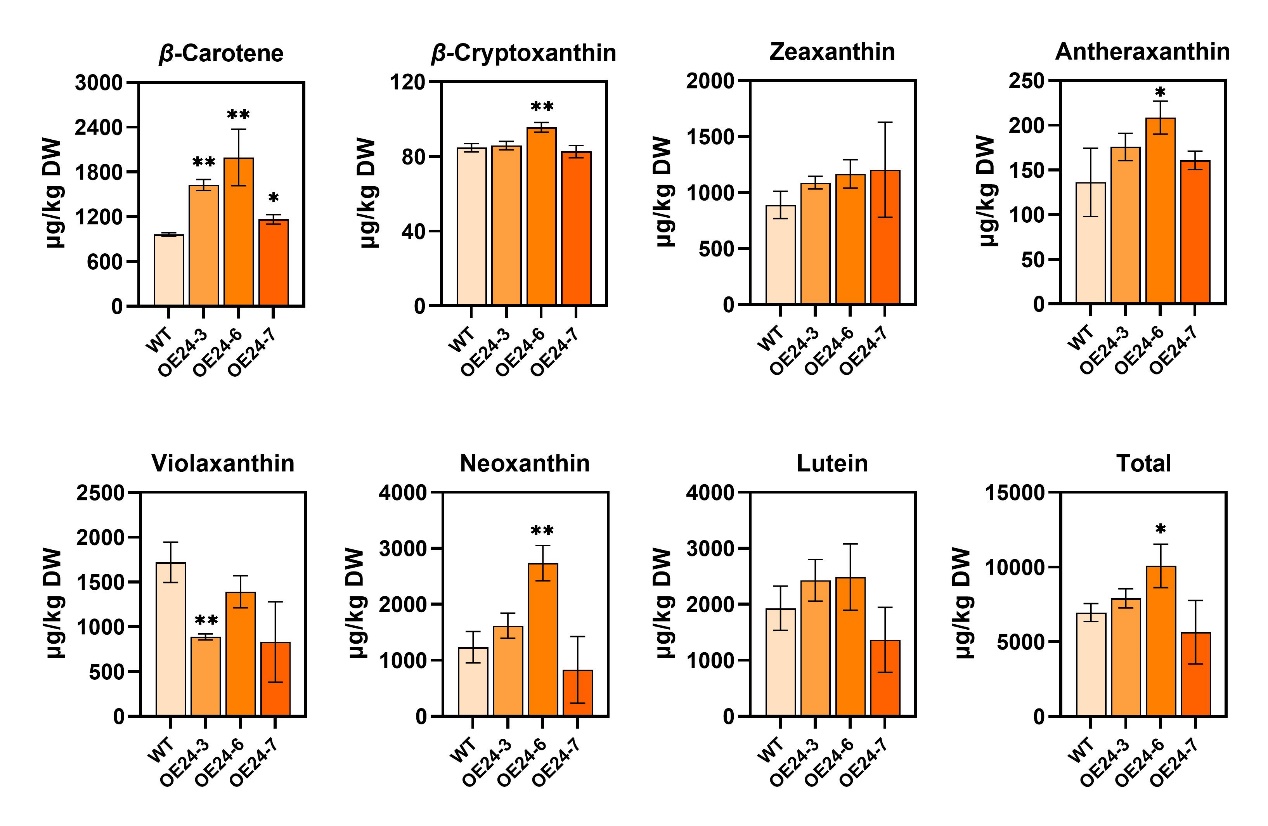


**Supplemental Figure S6.** The effects of VviWRKY24 on carotenoids accumulation in grape calli. DW, dry weight; WT was used as the control, error bars indicate means ± SD from three replicates, asterisks indicate significant differences by multiple *t*-tests; *, *p* < 0.05, **, *p* < 0.01.
